# Associations Between Children’s Numeracy Competencies, Mothers’ and Fathers’ Mathematical Beliefs, and Numeracy Activities at Home

**DOI:** 10.3389/fpsyg.2022.835433

**Published:** 2022-04-14

**Authors:** Anna Mues, Astrid Wirth, Efsun Birtwistle, Frank Niklas

**Affiliations:** Department of Psychology, Ludwig-Maximilians-Universität München, Munich, Germany

**Keywords:** parental beliefs, home numeracy environment, numeracy competencies, gender stereotypes, self-efficacy, importance of mathematical activities at home

## Abstract

Children’s numeracy competencies are not only relevant for their academic achievement, but also later in life. The development of early numeracy competencies is influenced by children’s learning environment. Here, the home numeracy environment (HNE) and parent’s own beliefs about mathematics play an important role for children’s numeracy competencies. However, only a few studies explicitly tested these associations separately for mothers and fathers. In our study, we assessed mothers’ and fathers’ mathematical gender stereotypes, self-efficacy and their beliefs on the importance of mathematical activities at home, and tested their associations with parents’ numeracy activities and children’s numeracy competencies in a sample of *N* = 160 children (*n* = 80 girls) with an average age of *M* = 59.15 months (*SD* = 4.05). Both, fathers and mothers regarded boys as being more competent in mathematics than girls. Fathers when compared to mothers reported a greater mathematical self-efficacy. Further, only mothers’ self-efficacy was associated with the frequency of numeracy activities with the study child. In contrast, only fathers’ beliefs on the importance of mathematics was associated with their numeracy activities which, in turn, predicted children’s numeracy competencies. However, the non-invariant constructs and varying results lead to the question whether a revision of existing scales assessing parental beliefs and home numeracy activities is needed to investigate differences of mothers and fathers and their potential associations with children’s numeracy outcomes.

## Introduction

Children’s early competencies and their development are supported by different experiences and aspects in their environment and in everyday life (e.g., Burghardt et al., 2020). In addition to kindergarten attendance (Melhuish et al., 2015) and the home learning environment (LeFevre et al., 2009; Anders et al., 2012; Niklas and Schneider, 2014), parents’ beliefs, attitudes and expectations (Sonnenschein et al., 2012; Skwarchuk et al., 2014; del Río et al., 2017) are discussed as important predictors of children’s early numeracy development.

Children’s early numeracy competencies are essential prerequisites for their later mathematics performance, academic achievement, and school success (Duncan et al., 2007; Jordan et al., 2007; Niklas and Schneider, 2017). Aspects such as child and family characteristics [e.g., sex or socioeconomic status (SES)] further influence children’s cognitive development (Niklas and Schneider, 2014).

The home numeracy environment (HNE) focuses on the early numeracy activities of parents and their children at home (LeFevre et al., 2009). Ecological and sociocultural theories emphasize the importance of the HNE for children’s mathematical development (Vygotsky, 1980; Bronfenbrenner and Morris, 2006). Recent studies support this association and reported a positive correlation between home numeracy activities and children’s numeracy competencies (LeFevre et al., 2009; Skwarchuk, 2009; Niklas and Schneider, 2014, 2017). However, additional relevant factors such as parental beliefs and expectations toward mathematics, have rarely been considered in recent research (del Río et al., 2017). Further, there are currently only a few studies (Tomasetto et al., 2015; del Río et al., 2019, 2020) that investigated potential differences between mothers and fathers, which may offer interesting insights as fathers have only recently become more involved in their children’s lives in many countries (Cabrera et al., 2000; Baker, 2014). The present study investigated children’s numeracy competencies and took the following factors into account; (1) three types of parental beliefs toward mathematics: gender stereotypes, self-efficacy and beliefs on the importance of mathematical activities at home, (2) differences in mothers’ and fathers’ beliefs toward mathematics and home numeracy activities, and (3) children’s sex.

### Children’s Early Numeracy Competencies

Children develop various numeracy competencies even before the start of their formal education (e.g., counting, number line estimation or knowledge of numbers and quantities; Krajewski and Schneider, 2009). Early development of academic competencies is highly predictive for success at school and later in life (Duncan et al., 2007; Jordan et al., 2007; Niklas and Schneider, 2017) as well as for children’s further mathematical development (Geary et al., 2009; Krajewski and Schneider, 2009; Jordan et al., 2010). However, these competencies vary greatly between children by the time they start school (Gould, 2012) and are influenced by diverse environmental aspects such as the home learning environment (LeFevre et al., 2009, 2010; Niklas and Schneider, 2017; Susperreguy et al., 2021) and parents’ beliefs and expectations toward mathematics (Skwarchuk et al., 2014; del Río et al., 2017). For boys and girls in contrast, often no significant differences concerning numeracy-related and language abilities were found at school entry (Niklas and Schneider, 2012; Kersey et al., 2018). Consequently, girls and boys seem to bring along more or less equal abilities at this age regardless of their sex.

Nguyen et al. (2016) suggested that early numeracy abilities are the strongest predictors of later mathematical achievement. Here, children’s advanced counting competencies (i.e., counting forward or backward from a given number or counting with cardinality) have been shown to be more predictive than their basic counting competencies (i.e., number recognition or verbal counting). This finding suggests that it is important to promote advanced counting activities and not to focus on basic counting skills only. However, basic skills still need to be considered as they build the basis for some advanced competencies and critical concepts. Further, children’s understanding of quantities and number words and arithmetic abilities were also identified as important predictors for later mathematical competencies (Jordan et al., 2006, 2009; Krajewski and Schneider, 2009). Our study investigated children’s numeracy outcomes in the context of additional potentially influencing factors (e.g., HNE and parents’ characteristics) to elaborate which aspects play an important role for children’s numeracy development before developing later mathematical skills.

### Home Numeracy Environment and Parental Involvement in Mathematical Outcomes

The HNE is defined as the interaction between children and their parents concerning numeracy activities within the home environment (e.g., playing dice or counting games and exposure to numerical content; e.g., LeFevre et al., 2009; Skwarchuk et al., 2014). The HNE may be differentiated into formal and informal home activities (e.g., LeFevre et al., 2009). Here, aspects such as using number books and active stimulation of number skills, which require dynamic engagement and the intention of parents to teach mathematics to their children are subsumed as formal HNE. In contrast, informal aspects were described as activities that ‘incidentally’ support children’s numeracy abilities such as naturally occurring activities in the home that induce counting or exposure to numbers (e.g., playing mathematical games and involving the child in measuring ingredients).

Playful learning activities motivate and engage children in learning numbers, counting, and reasoning, and also prepare them to advance their mathematical thinking skills (e.g., problem solving, mental representation of numbers; Cohrssen et al., 2014; Niklas et al., 2016). Parents often provide such learning opportunities and thus motivate their children to learn mathematics (Cohrssen and Niklas, 2019; Gasteiger and Moeller, 2021). For example, Cohrssen and Niklas (2019) reported gains in children’s mathematical competencies through playing a math game, underlining the importance of parent-child interactions in shared mathematical activities.

Further, LeFevre et al. (2009) showed that children who are often involved in mathematical activities with their parents at home, are more likely to improve their computational efficacy and accuracy while solving mathematical problems (see also Kleemans et al., 2012). However, positive associations (Niklas and Schneider, 2014; Skwarchuk et al., 2014; Susperreguy et al., 2020) and no significant associations (Skwarchuk, 2009; DeFlorio and Beliakoff, 2015; Missall et al., 2015) were found for the formal and the informal HNE and children’s mathematical outcomes with the informal HNE seemingly being the better predictor (LeFevre et al., 2009). Consequently, more research on the specific aspects and mechanisms that may support children’s mathematical learning in the context of the HNE are necessary (see also Hornburg et al., 2021).

For instance, research identified additional parental factors which can be linked to the HNE and may impact on children’s competencies such as parental beliefs, attitudes and expectations (Sonnenschein et al., 2012; Skwarchuk et al., 2014; del Río et al., 2017). It is also of interest, whether numeracy activities at home and children’s numeracy outcomes are influenced by further parental factors such as potential differences between mothers and fathers.

### Parental Beliefs Toward Mathematics

Research indicates that parental beliefs, expectations and attitudes predict parental numeracy activities and children’s numeracy competency development (Skwarchuk et al., 2014; Missall et al., 2015; del Río et al., 2017; Susperreguy et al., 2020), which aligns with the expectancy-value theory by Eccles. This theory assumes that parental beliefs influence children’s achievement motivation, their educational aspirations, and their abilities and provide them with experiences at home and in everyday life which are directed by the beliefs of the parents (Eccles et al., 1983; Jacobs et al., 2005). Recent studies also suggest a direct link between parental beliefs, children’s self-concept and their mathematical performance (del Río et al., 2019, 2020), indicating that parents’ personal beliefs and thoughts may have a tremendous impact on children’s perception of their own abilities and thus also on children’s academic outcomes in mathematics.

Parental beliefs toward mathematics can simply be defined as parents’ interest in mathematics and their feeling of confidence while performing mathematics (Benz, 2012). Further constructs such as gender stereotypes, self-efficacy and the importance of mathematical activities at home are often subsumed under the umbrella-term *beliefs* (e.g., Sonnenschein et al., 2012, 2016; del Río et al., 2017, 2020). Research shows that parents who tend to have positive beliefs regarding mathematics also engage more frequently in formal numeracy practices such as counting pieces of a pie and teaching how to count (Skwarchuk et al., 2014; Missall et al., 2015; del Río et al., 2017).

Further, parents who engage in formal numeracy activities frequently and who enjoy doing mathematics were reported to have higher expectations for both themselves and their children to perform successfully in numeracy tasks (Blevins-Knabe et al., 2000; Kleemans et al., 2012; Skwarchuk et al., 2014; del Río et al., 2017). However, the associations between parental beliefs and expectations, numeracy-related activities at home and children’s numeracy outcomes seem to vary between studies (LeFevre et al., 2009; Skwarchuk, 2009; Sonnenschein et al., 2012; del Río et al., 2017). This finding inspired us to investigate the role that parental beliefs toward mathematics (especially gender stereotypes, self-efficacy and the importance of mathematical activities at home) play for parents’ numeracy practices and children’s early numeracy competencies.

### Gender Stereotypes

Gender stereotypes in certain academic areas such as mathematics are often observed in society and they may differ across countries and cultures (Nosek et al., 2009; Breda et al., 2020; Lewis and Lupyan, 2020). As an important part of the societal structure, parents tend to have stereotypes concerning gender and occupation (Breda et al., 2020; del Río et al., 2020). These beliefs are not necessarily developed intentionally, however, they still may impact on children’s own beliefs about mathematics and their actual outcomes (Sonnenschein et al., 2012; del Río et al., 2019, 2020). Gender stereotypes in mathematics can simply be described as favoring one gender over another (e.g., boys can do mathematics better than girls, del Río et al., 2020).

In their systematic review, Gunderson et al. (2012) showed that parents’ gender stereotypes and their expectations directly affected children’s own beliefs, success and achievement in mathematics. These stereotypes do not only impact on their children’s own beliefs and development, they also influence how parents engage with their children while doing mathematical activities. For instance, parents tend to engage with sons more often than with daughters (Jacobs et al., 2005; Nosek et al., 2009; Gunderson et al., 2012; del Río et al., 2017). Moreover, del Río et al. (2017) reported that mothers’ engagement in advanced numeracy activities differed depending on the sex of their child; that is, mothers engaged with boys more often than with girls. Consequently, recent research indicates that parental gender stereotypes seem to impact on parent-child interactions as well as on children’s achievement and development.

### Self-Efficacy

Parents’ mathematical self-efficacy also plays an important role for their mathematical beliefs, their own mathematical experiences and achievements, and the mathematical interactions with their children (Missall et al., 2015). Self-efficacy describes the interest in and the ability to achieve certain behaviors successfully (Bandura, 1977). Parental mathematical self-efficacy can thus be defined as parents’ belief of being able to solve mathematical problems and their belief of being able to influence children’s mathematical learning and their environment in a supportive way (Ardelt and Eccles, 2001).

Peacock-Chambers et al. (2017) showed that high levels of parental self-efficacy were associated with a better quality home learning environment. Further, the frequency of informal mathematical activities and its association with children’s numerical understanding was mediated by parents’ mathematical self-efficacy and their attitudes toward mathematics. Here, parents’ mathematical self-efficacy was also linked indirectly to children’s arithmetic skills via informal mathematical activities (Vasilyeva et al., 2018).

### Parental Beliefs on the Importance of Mathematical Activities

Parental beliefs about the importance of doing mathematical activities at home are regarded as another important factor that is associated with the HNE and children’s engagement in mathematical activities (e.g., Sonnenschein et al., 2012). Sonnenschein et al. (2012) analyzed parental beliefs on the importance of mathematical activities at home and their relation with children’s mathematical activities at home. Most of the surveyed parents regarded mathematics at home to be very important and only 14% of the parents considered it as being not so important.

Parents who reported mathematical activities at home to be important, not only had a more positive attitude toward supporting children’s mathematical learning, their children also engaged in mathematical activities at home more often (Sonnenschein et al., 2012).

Although the HNE and parental beliefs are associated with children’s numeracy competencies, we still do not know much about potential differences between the beliefs of mothers and fathers, and whether they may be associated differentially to children’s numeracy skills.

### Differences in Mothers’ and Fathers’ Mathematical Beliefs and the Numeracy-Related Interactions With Their Child

Previous research on parents’ numeracy activities and the interactions with their children has usually relied on data reported by mothers only (Saracho and Spodek, 2008). However, there is some research that took both mothers and fathers into account. For instance, del Río et al. (2017) showed that mothers’ advanced numeracy-related interactions were a better predictor for children’s numeracy outcomes than fathers’ interactions, suggesting that mother-child and father-child interactions may support children’s learning in different ways. Further, an indirect effect of mothers’ expectations toward mathematics and children’s numeracy outcomes was found through the advanced numeracy activities mothers provided at home, whilst no such association was detected for fathers’ expectations.

Tomasetto et al. (2015) reported a specific role of mothers’ math-gender stereotypes concerning their daughters, but not their sons. del Río et al. (2020) further reported that mothers’ and fathers’ implicit measures both showed a stronger association for mathematics with males than females. Significantly stronger math-gender stereotypes were found for the explicit measures of mothers compared to fathers. In addition, fathers compared to mothers were more convinced that they are good in mathematics.

The scarcity of research that focusses on the differences between mothers and fathers in their beliefs and numeracy activities at home further underlines the need for such studies that examine the relation of these aspects with children’s numeracy outcomes.

#### The Present Study

In recent years, several studies analyzed the home numeracy activities and parental beliefs (e.g., del Río et al., 2017; Susperreguy et al., 2020). However, only a few studies investigated mothers’ and fathers’ beliefs toward mathematics and potential differences (e.g., Tomasetto et al., 2015; del Río et al., 2017). In addition, few studies considered the associations between children’s sex, children’s numerical competencies and the HNE simultaneously (del Río et al., 2017, 2020). Therefore, in the present study, we will try to identify aspects that influence children’s numeracy competencies. Here, we analyze three types of parents’ mathematical beliefs—namely gender-stereotypes, self-efficacy, and parental beliefs on the importance of mathematical activities at home—and the numeracy practices they conduct with their children at home. One key objective is to investigate potential differences in mothers’ and fathers’ beliefs toward mathematics and their numeracy activities at home and children’s numeracy competencies while considering both parents’ and children’s sex.

Accordingly, we were interested in answering the following four questions:

(1) Do we find measurement invariance for our constructs, when we ask mothers and fathers the same questions?
(2) Do we find differences between mothers’ and fathers’ beliefs toward mathematics (i.e., concerning gender stereotypes, self-efficacy, and beliefs on the importance of mathematical activities at home)?
(3) Is there an association between these aspects and the numeracy-related activities at home and children’s numeracy outcomes?
(4) Do these associations differ for boys and girls?

To answer these questions, we tested the following five hypotheses:

(1) We expected to measure the same constructs (i.e., beliefs and HNE), when we ask mothers and fathers the same questions (i.e., measurement invariance).
(2) We suggest that mothers and fathers will differ significantly in their mathematical gender stereotypes, self-efficacy and the reported importance of mathematical activities at home. Here, we expected mothers to show lower mathematical self-efficacy than fathers (del Río et al., 2019, 2020).
(3) In addition, we hypothesized that mothers who expect boys to excel in mathematics when compared to girls to have a lower mathematical self-efficacy, whereas fathers with the same stereotypes should have a higher mathematical self-efficacy (del Río et al., 2019, 2020).
(4) We expected parents with a greater mathematical self-efficacy who reported less strong gender stereotypes toward mathematics to engage more often in numeracy related activities and to have children who perform better in the numeracy tasks (del Río et al., 2017).
(5) Finally, we expected that the findings and associations will differ significantly dependent on whether the study child is a boy or a girl.

## Materials and Methods

### Sample

We assessed children’s numeracy competencies (NumC) in a sample of *N* = 310 children (*n* = 160 girls) with an average age of *M* = 59.36 months (*SD* = 3.94) and surveyed both, their mothers and fathers concerning their mathematical self-efficacy (SE), gender stereotypes (GS), beliefs on the importance of mathematical activities at home (IOMA), and the numeracy activities they provide at home to their children (NA). The data was taken from the first measurement point of the second cohort of the EU-funded, 5-year-longitudinal study “Learning4Kids” project (Niklas et al., 2020a). Trained psychologists, educators and research assistants performed the assessments which included standardized numeracy tests to assess children’s numeracy competencies. Further, parents were asked to fill in a written survey assessing SE, GS, IOMA, NA, their family background and children’s characteristics. Here, children’s sex assigned at birth was reported by their parents in our parental survey.

The majority of our sample spoke German as main language (68.1%). Families, whose first language was not German (27.5%) reported 16 different languages as main language and were provided with surveys in their own language when possible (e.g., Turkish, Polish, English, etc.). Before the beginning of the assessments, families were contacted via mail and received a description of the study and the invitation to contact the project team for participation via e-mail or telephone. In a next step, we called all the families who indicated their interest to participate in our study and explained the study requirements and obtained an informal consent. Some of the participating families were recruited through kindergartens. During the family visit, formal consents were collected. All research activities were approved by the European Research Council Executive Agency and the Ethics committee of the Faculty of Psychology and Educational Sciences at the University of Munich.

As our research focuses on differences between fathers’ and mothers’ mathematical beliefs and interactions and as the great majority of children in our sample lived together with both parents, only children for whom data from both, mothers and fathers were available, were included in the analyses.^1^ Accordingly, about half of the sample (children with complete mother-father dyads) were included in the analytic sample (*N* = 160, *n* = 80 girls). Children in this subsample had an average age of *M* = 59.15 months (*SD* = 4.05). A potentially biased drop-out between the excluded and all other cases was tested with independent *t*-tests for our study variables (i.e., numeracy activities, numeracy competencies, beliefs, SES, age, sex). No significant differences between the excluded and all other cases were found (all *p*’s > 0.05, *BF*_10_ < 0.07), except for the beliefs on the IOMA of fathers which were significant (*p* < 0.05), but the Bayes Factor was low (*BF*_10_ = 1.37), indicating that the analytic sample seems to be comparable to the total sample.

### Measures

#### Children’s Numeracy Competencies

All participating children were assessed with various numeracy tests. We used the “Marko-Screening—mathematics and concepts of calculation before school entry” (MARKO-S; Ehlert et al., 2020) which includes 21 items concerning numbers, cardinality, ordinal number bars and number division, inclusion and relations (Cronbach’s α = 0.78 and Mc Donald’s ώ = 0.77). Further, addition and subtraction were tested by an adapted version of the calculation subtest of the “Assessment of basic mathematical competencies in kindergarten” (Krajewski, 2018) with eight items (Cronbach’s α = 0.70 and Mc Donald’s ώ = 0.69). Various subtests from the “Würzburger preschool test: Assessments of literacy and mathematical (precursor) abilities and linguistic competencies in the last year of kindergarten” (Endlich et al., 2017) were applied to assess competencies such as number sequences forward, number sequences backward, number symbol knowledge and knowledge of numerical representations (Cronbach’s α = 0.92 and Mc Donald’s ώ = 0.92). All of the subtests consisted of eight items, except number sequences backward with six and knowledge of numerical representations with 10 items. Afterward, scales were built from all items for each subtest. Finally, children’s numeracy competencies were measured by a latent variable including all numeracy items (Cronbach’s α = 0.93 and Mc Donald’s ώ = 0.93).

#### Parental Surveys

Both parents completed our surveys (see survey questions in Supplementary Material). The caregiver who was present during the assessments was asked to fill in the main questionnaire which consisted of questions regarding numeracy practices provided at home, family and child characteristics and additionally included questions about the own beliefs toward mathematics. The other parent, who was or was not present during the assessments, was also asked to fill in a survey which only consisted of questions on home numeracy activities and beliefs toward mathematics. The main questionnaire was offered as a paper and pencil survey at home, whereas the additional parental survey was offered either as a paper and pencil survey version or as an online survey version, to assess as many parental pairs as possible.

#### Numeracy Activities at Home

Parents were asked about informal numeracy-related activities that they do together at home with their children. The NA were measured as a latent variable and contained six items (adapted from Niklas et al., 2016) with questions about parents’ involvement in everyday numeracy activities [e.g., “How often do you involve your child in cooking (e.g., counting, weighing, or measuring ingredients)?] (Mothers’ Cronbach’s α = 0.65 and Mc Donald’s ώ = 0.68; Fathers’ Cronbach’s α = 0.67 and Mc Donald’s ώ = 0.65). Parents rated the items on a 5-point Likert scale (e.g., from several times a week to never). Values of 4–0 were assigned accordingly with higher values indicating more frequent NAs and the values were averaged for both, fathers and mothers.

#### Parental Beliefs

Parental beliefs toward mathematics were assessed with statements concerning parents’ own mathematical SE, their GS toward mathematics and the IOMA at home. Parental SE was measured with 6 items (Mothers’ Cronbach’s α = 0.75 and Mc Donald’s ώ = 0.87; Fathers’ Cronbach’s α = 0.77 and Mc Donald’s ώ = 0.87) and included statements such as “In school, I was good at math” (see also Skwarchuk et al., 2014; Missall et al., 2015; Susperreguy et al., 2020). Parental GS were surveyed with three items (e.g., “Girls need less assistance than boys in mathematics”) (Mothers’ Cronbach’s α. = 87 and Mc Donald’s ώ = 0.87; Fathers’ Cronbach’s α = 0.88 and Mc Donald’s ώ = 0.89), that are based on work by Tatto et al. (2012), Tomasetto et al. (2015), and Blömeke et al. (2017). Parents were also asked to evaluate the importance of their child doing mathematical activities at home with three items (e.g., “It is important to me that my child does mathematical activities at home”) (Mothers’ Cronbach’s α = 0.50 and Mc Donald’s ώ = 0.50; Fathers’ Cronbach’s α = 0.62 and Mc Donald’s ώ = 0.62) (Sonnenschein et al., 2012). These items were adapted for our study and values of 0–4 were assigned (from “not at all true” to “completely true”).

### Statistical Analysis

Data analysis was performed by using IBM SPSS Statistics 28.0 (IBM Corp, 2021), JASP 0.16.0.0 (JASP Team, 2021) and Mplus 8.7 (Muthen and Muthen, 2021). Bayes factors were calculated with JASP (Overstall and King, 2014; Morey and Rouder, 2015; JASP Team, 2021).

The percentage of missing values at the item level of childrens’ variables as well as mothers’ and fathers’ variables was low (max. 8.1%). Children’s missing values ranged from 0.6 to 8.1%. Mothers had a range of missing values from 1.2 to 3.1% and for fathers, missing values ranged from 0.6 to 1.2%.

First, a multiple-group confirmatory factor analysis (MGCFA) was conducted to test our analyzed constructs for mothers and fathers. Here, we implemented the diagonally weighted least square estimator (DWLS, WLSMV in MPLUS) as this estimator is recommended to be used for categorical ordinal data and its usage leads to more reliable results when ordered Likert scales are applied (Li, 2016, 2021; Lionetti et al., 2016). Next, we tested measurement invariance to check the comparability of the constructs for mothers and fathers (see Supplementary Material).

**FIGURE 1:**
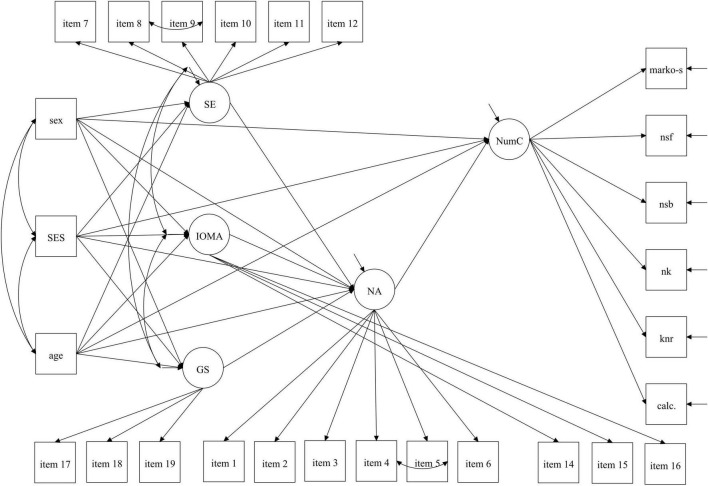
Modified theoretical Model of the associations between parents’ beliefs, their numeracy activities and children’s numeracy competencies. NumC, children’s numeracy competencies; NA, numeracy activities at home; GS, gender stereotypes; SE, self-efficacy; IOMA, importance of mathematical activities at home. Sex, children’s sex; Age, children’s age in month. marko-s, Marko-S; nsf, number sequences forward; nsb, number sequences backward; nk, number knowledge; knr, knowledge of number representation; calc. calculation task. Item description of items 1–19 (see Supplementary Material).

Descriptive statistics of parents’ beliefs toward mathematics and their numeracy activities are shown in Table 1. Bayesian paired *t*-tests as well as Bayesian repeated-measurement analyses of variance (ANOVA) were applied to test for potential differences between mothers’ and fathers’ beliefs, their NA, and to check whether and how parents’ mean values may vary for boys and girls. Further, Bayesian independent *t*-tests were conducted to investigate how mothers’ and fathers’ beliefs differ when having a son or a daughter as study child. Additionally, we tested mothers’ and fathers’ values in GS and the reported IOMA against an expected mean value by using Bayesian one-sample *t*-tests. Here, parents’ answers were reported on a 5-point Likert scale from 0 to 4. The expected mean value of 2 for GS would indicate no perceived differences between boys’ and girls’ mathematical abilities. These analyses are based on the theoretical assumption that boys and girls at kindergarten age do not differ in regard to their numerical abilities (Niklas and Schneider, 2012; Kersey et al., 2018). Consequently, we assume that parents should not attribute more competence to one sex over the other (boys vs. girls). For IOMA, the expected mean value of 2 would indicate, that parents’ regard mathematical activities at home neither as important nor as unimportant. Here, we expected parents would regard the IOMA on average above the mean value of 2 (see Sonnenschein et al., 2012). Finally, a multiple-group structural equation model (MGSEM) was used to analyze the associations of our theoretical model (see Figure 1). To evaluate the model fit, several goodness-of-fit indices were considered: the root mean square error of approximation (RMSEA, ≤ 0.06), the comparative fit index (CFI, ≥ 0.95), and the standardized root mean squared residuals (SRMR, ≤ 0.08) (Hu and Bentler, 1999). Although we also report the Chi-Square goodness-of-fit statistic (*X*^2^, *p* ≥ 0.05), this measure may be oversensitive to minor model misspecifications and sample size (Chen, 2007). Modification indices aligning with theory were considered to improve the model fit. Here, the highest modification indices one after another were added to the model to examine the changes until a sufficient model fit was achieved (Schumacker and Lomax, 2010). All applied modifications are described in our “Results” section.

**TABLE 1:** Descriptive statistics of parental variables for the total analytic sample and subsamples of boys and girls.

	Total					Boys					Girls				
	*N*	Min	Max	*M*	*SD*	*N*	Min	Max	*M*	*SD*	*N*	Min	Max	*M*	*SD*
NA m	160	0.67	4.00	2.27	0.76	80	0.67	3.67	2.27	0.75	80	0.67	4.00	2.27	0.78
NA f	160	0.17	3.83	2.18	0.74	80	0.17	3.83	2.09	0.74	80	0.67	3.67	2.27	0.73
GS m	156	0.00	3.33	1.47	0.76	78	0.00	3.00	1.41	0.73	78	0.00	3.33	1.54	0.78
GS f	159	0.00	3.33	1.36	0.87	79	0.00	2.33	1.23	0.84	80	0.00	3.33	1.49	0.89
SE m	159	0.00	4.00	2.83	0.99	80	0.00	4.00	2.84	0.94	79	0.00	4.00	2.81	1.05
SE f	160	0.20	4.00	3.13	0.85	80	0.20	4.00	3.05	0.86	80	0.60	4.00	3.20	0.85
IOMA m	157	1.00	4.00	2.94	0.66	79	1.33	4.00	2.84	0.66	78	1.00	4.00	3.03	0.66
IOMA f	159	0.33	4.00	2.81	0.74	79	0.33	4.00	2.70	0.78	80	0.67	4.00	2.91	0.68

*N, sample size; Min, minimum; Max, maximum; M, mean; SD, standard deviation; NA, numeracy activities; GS, gender stereotypes; SE, self-efficacy; IOMA, importance of mathematical activities at home; m, mothers; f, fathers.*

## Results

### Construct Validity and Measurement Invariance

First, we evaluated our measurement models for the study variables using MGCFA. The model fit of the theoretical model was good, except for the SRMR which showed a value slightly above the cut-off [*X*^2^(556) = 1024.019, *p* < 0.01, RMSEA = 0.07, CFI = 0.93, SRMR = 0.08]. To improve the model fit, some modification indices as suggested by MPlus were included after careful theoretical considerations. Here, correlations of item residuals were included by using the WITH statement of MPlus. For mothers’ and fathers’ numeracy activities, a correlation of the item residuals of item 4 with item 5 was included (see item description in Supplementary Material). Further, for parent’s self-efficacy, correlations of the item residuals of items 9 and 10 were included (see item description in Supplementary Material). With the application of these modification indices, our model showed a slightly better model fit [*X*^2^(552) = 929.284, *p* < 0.01, RMSEA = 0.07, CFI = 0.95, SRMR = 0.08]. The Chi-square differentiation test showed a significant *p*-value (*X*^2^ = 124.007, *df* = 4, *p* < 0.001), indicating that we can proceed with this modified model.

In order to evaluate whether we assessed equal constructs for mothers and fathers with our parental survey, measurement invariance was tested. For later analyses (i.e., paired *t*-tests, and MGSEM), scalar invariance was needed to compare the latent means of mothers and fathers. To evaluate the model fit of the observed data, the change of the alternative Comparative Fit Index (CFI; ≤ –0.01) and root mean square error of approximation (RMSEA; ≤ 0.015) was used instead of the very sensitive Chi-Square (*X*^2^) (Chen, 2007). For our measurement model, configural invariance was found only. This finding indicates that the results of mothers and fathers cannot be compared in regard to mean difference tests, but that our survey questions seem to measure the same factor structure of our constructs for mothers and fathers (see Supplementary Material). We will continue with the planned comparisons between mothers and fathers, but will discuss this limitation later.

### Mothers’ and Fathers’ Beliefs and Home Numeracy Activities

Paired *t*-tests showed that no significant differences between mothers’ and fathers’ beliefs and numeracy activities were found, with the exception of SE. Here, fathers showed a significantly greater SE toward mathematics than mothers [fathers: *M* = 3.13, *SD* = 0.85; mothers: *M* = 2.83, *SD* = 0.99; *t*(158) = –3.08; *p* < 0.01; *BF*_10_ = 8.14, Cohen’s *d* = –0.24, small effect size]. Further, no significant differences for mothers’ and fathers’ beliefs and numeracy activities were found when the study child’s sex was included in our repeated-measurement ANOVAs. Consequently, no differences for mothers and fathers were found, independent of the sex of the study child.

Further, parents were asked, whether girls have better mathematical competencies and need less support than boys. On average, both mothers and fathers regarded boys to be more competent in mathematics than girls [comparison to expected mean: mothers: *t*(155) = –8.68, *p* < 0.01, Log(*BF*_10_) = 27.83, Cohen’s *d* = –0.70, medium effect size; fathers: *t*(158) = –9.261, *p* < 0.01, Log(*BF*_10_) = 31.34, Cohen’s *d* = –0.73, medium effect size]. In addition, parents regarded mathematical activities at home to be important on average [comparison to the expected mean: mothers: *t*(156) = 17,679, *p* < 0.01, Log(*BF*_10_) = 82.33, Cohen’s *d* = 1.1, large effect size; fathers: *t*(158) = 13.825, *p* < 0.01, Log(*BF*_10_) = 59.40, Cohen’s *d* = 1.41, large effect size].

### Associations Between Parents’ Beliefs Toward Mathematics, Their Numeracy Activities and Children’s Numeracy Competencies

**FIGURE 2:**
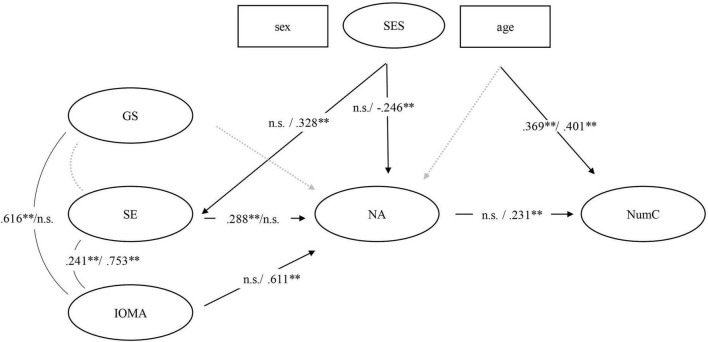
Path analysis of the associations between mothers’ and fathers’ beliefs toward mathematics, their numeracy activities at home, and numeracy competencies of the children. NumC, children‘s numeracy competencies; NA, numeracy activities at home; GS, gender stereotypes; SE, self-efficacy; IOMA, importance of mathematical activities at home. Sex, children’s sex; Age, children’s age in month. Black lines indicate significant associations, and dotted gray lines indicate non-significant associations. ^**^p < 0.01, n.s., non-significant. Path coefficients for mothers/fathers.

To evaluate the associations between our study variables, a MGSEM was conducted (see Figure 2).

#### Path Analysis

In our model, we expected a direct association between parents’ NA and NumC and an indirect effect of mothers’ and fathers’ beliefs toward mathematics on children’s NumC via the NA. Further, we controlled for children’s sex, age and families’ SES. We used a MGSEM to compare mothers and fathers (see Figure 1). Here, the goodness-of-fit indices suggested a good model fit, with the exception of the SRMR [*X*^2^(676) = 864.049, *p* < 0.001, RMSEA = 0.04, CFI = 0.97, SRMR = 0.11].

Results from our MGSEM demonstrated that mothers’ NA were not significantly (*β* = 0.096, *p* > 0.05) associated with children’s NumC (see Figure 2). The indirect paths of mothers’ beliefs on children’s NumC via NA were also not significant. However, mothers’ reported SE was significantly positively associated with their NA (*β* = 0.319, *p* < 0.001). In addition, mothers’ IOMA and GS (*β* = 0.616, *p* < 0.001) and IOMA and SE (*β* = 0.251, *p* < 0.01) were associated, indicating that the attitude of the importance to do mathematical activities at home is accompanied by a more positive attitude that girls are more competent in mathematics than boys. No significant association was found between their GS and their SE. Additionally, children’s age was significantly associated with children’s NumC (*β* = 0.367, *p* < 0.001). Here, older children had better outcomes in comparison to younger children. No significant associations were found for children’s sex and families SES and mothers’ beliefs and NA.

For fathers, we found a significant association between their NA and children’s NumC (*β* = 0.251, *p* < 0.01). Contrary to mothers, fathers’ beliefs on the IOMA were significantly positively associated with their NA (*β* = 0.611, *p* < 0.01). Here, additionally a total indirect effect on children’s NumC was found (*β* = 0.153, *p* < 0.05), revealing that fathers who value the importance of mathematical activities to a greater extent also engage more often in numeracy activities at home with their children, who—in turn—show better numeracy abilities. No further indirect effects were found for fathers’ beliefs, their NA and NumC. Their SE and GS were not significantly associated with each other and with the NA. However, there was a significant correlation between fathers’ SE and their beliefs on the IOMA (*β* = 0.774, *p* < 0.001), whereas no such association was found for GS and IOMA. In regard to our control variables, SES was positively associated with fathers’ SE (*β* = 0.338, *p* < 0.001) and negatively associated with their NA (*β* = –0.243, *p* < 0.05). Again, children’s age was associated with children’s NumC (*β* = 0.401, *p* < 0.001). No significant associations were found for children’s sex and the other study variables.

## Discussion

Children’s early numeracy competency development and later mathematical achievement have been investigated previously, however, many questions about influencing factors such as the HNE or parental beliefs remained unanswered (Niklas and Schneider, 2014, 2017; del Río et al., 2017; Susperreguy et al., 2021). This study investigated potential differences between mothers’ and fathers’ beliefs and numeracy practices at home. Contrary to recent research (Niklas and Schneider, 2014, 2017; Skwarchuk et al., 2014; del Río et al., 2017; Susperreguy et al., 2020), our results only confirmed a significant association of fathers’ numeracy practices at home and children’s numeracy competencies, but not of mothers’ numeracy practices and children’s numeracy competencies. Moreover, our results expand current research (del Río et al., 2020; Susperreguy et al., 2020) by contributing to the identification of further factors that may influence children’s numeracy competency acquisition and parental practices by examining the distinct associations of mothers’ and fathers’ beliefs and child outcomes.

The analyses of measurement invariance (H1) showed that the questions we asked mothers and fathers may have assessed the same factor structure of our constructs for mothers and fathers, however, the means were not equivalent. Here, it may be possible that mothers and fathers have a different understanding of our constructs, as found by Meunier and Roskam (2009) who analyzed self-report self-efficacy and found differing associations for mothers and fathers. Consequently, more research is needed to develop standardized parental surveys that provide not only objective, reliable and valid assessments of parental beliefs, but that are also comparable across main caregivers. Such surveys should include questions about the formal and the informal HNE and may also refer to a broader construct of the home math environment by including further aspects of mathematics as proposed by recent literature (e.g., geometry, spatial activities, patterning, and measurement; Zippert and Rittle-Johnson, 2020; Hornburg et al., 2021). As no scalar measurement invariance was found, our reported findings must be interpreted with caution: No direct comparisons of the values of mothers and fathers are possible. Nevertheless, in the following we try to identify possible explanations for the potential differences we found between mothers and fathers.

Our results showed significant differences between mothers’ and fathers’ beliefs for parents’ self-efficacy (H2). As expected, fathers’ reported mathematical self-efficacy was greater than that of mothers (Hofmann et al., 2005; del Río et al., 2019, 2020). Here, societal and cultural stereotypes might have differential effects on males and females, as, for example, even if women work in STEM-related occupations, they still show lower self-concept scores in mathematics compared to men (Niepel et al., 2019; Breda et al., 2020; Lewis and Lupyan, 2020).

Another possible explanation could be that mothers seem to have a higher level of math anxiety than fathers, which is reflected in their self-assessment. For example, Schmader et al. (2004) showed that stereotypes can cause anxiety, which in turn has a negative effect on the awareness of one’s own abilities. Further, del Río et al. (2017) reported statistically significant higher math anxiety levels for mothers than for fathers, which led to less frequent engagement in mathematical activities. According to Hornburg et al. (2021), cultural influences need to be considered and more research is needed to identify mechanisms that lead to the on average lower SE of mothers. Moreover, as parental attitudes and believes may impact parents’ interactions with their children and children’s own attitudes (e.g., Niklas et al., 2020b), it is important to inform parents, especially mothers, and to intervene early so that any potential detrimental effects for children can be prevented.

Consistent with prior research, fathers and mothers in our sample regarded boys to be more competent in mathematics than girls (del Río et al., 2019, 2020). These results fit with the literature on mathematical gender stereotypes of adults (Nosek et al., 2009; Miller et al., 2015; Breda et al., 2020) and underline the suggestion of del Río et al. (2017), that cultural stereotypes may implicitly influence parents’ own experience of raising their own son or daughter.

As stated before and contrary to current research (Niklas and Schneider, 2014, 2017; Skwarchuk et al., 2014), our findings supported the assumption of a direct association of children’s numeracy competencies and parents’ numeracy-related activities for fathers only, but not for mothers. This finding stands in contrast to results of del Río et al. (2017), who reported a significant association for mothers only. Here, it should be noted that we only measured informal aspects of the HNE (see LeFevre et al., 2009) and that the associations reported by del Río et al. (2017) were found in the context of formal numeracy practices.

Focusing on the formal HNE or using a more comprehensive assessment of the home math environment as suggested in recent literature (e.g., Zippert and Rittle-Johnson, 2020; Hornburg et al., 2021) may lead to more informative and possibly different results. Such an approach may also help to shed light on the inconsistent findings concerning the association of formal and informal numeracy practices with children’s numeracy development (see Elliott and Bachman, 2018). Indeed, analyses with similar measures, but with data from only one main caregiver (mostly mothers) for whom more information about the HNE were assessed, showed that numeracy practices provided at home were a significant predictor of children’s numeracy competencies (Mues et al., 2021).

In regard to further associations of parental beliefs, numeracy activities and children’s numeracy competencies (H3 and H4), our findings showed that mothers with higher SE provided more frequent numeracy activities at home for their children. Similar results have been reported by Peacock-Chambers et al. (2017), who demonstrated that higher parental self-efficacy levels were associated with higher scores in measures of the home learning environment. This would also inversely be in line with findings of del Río et al. (2019), who reported that mothers with lower self-efficacy values provided lower quality numeracy activities for their children. Further, Vasilyeva et al. (2018) reported an indirect effect of parents’ self-efficacy to children’s arithmetic skills via parents’ informal mathematical activities.

Additionally, mothers’ reported IOMA and SE were significantly correlated. This result indicates that higher SE values and the reported IOMA may be mutually dependent (see also Sonnenschein et al., 2012). Our findings in regard to parental beliefs on the importance of doing mathematical activities at home are in line with findings from Sonnenschein et al. (2012, 2016) and demonstrate that both, mothers and fathers endorse the importance of mathematical activities at home.

In contrast to mothers, a significant association between the IOMA and fathers’ NA was found, indicating that the reported belief about the importance of numeracy activities at home is associated with the direct implementation of such activities. We further found an indirect effect of IOMA via NA on children’s numeracy abilities. This result aligns with findings from Sonnenschein et al. (2012), who found comparable results, when measuring parental beliefs and the frequency of children’s numeracy related activities. The higher SE of fathers compared to mothers may play a role for the different associations found for both main caregivers. Although no direct association of fathers’ SE with fathers’ NA were found, SE correlated significantly with fathers’ reported IOMA. Here, further research is needed to understand the causal associations of our measures and why specific associations were found for mothers or fathers only.

Children’s sex, did not show any significant associations with parental beliefs, NA, and child outcomes in any of our analyses (H5; see also De Keyser et al., 2020; Zippert and Rittle-Johnson, 2020). In contrast, for families’ SES, significant associations with fathers’ SE and NA were found, indicating higher SES is associated with a higher SE of fathers, but lower frequencies of NA. Tazouti and Jarlégan (2019) reported similar findings about the positive association between parents’ SES and self-efficacy, but stated that this association was stronger for mothers, contrary to our findings. However, in their analyses, they did not find measurement invariance, and therefore their results must be interpreted with caution.

As fathers are still considered as the main earner in families in many cultural contexts, societal expectations may influence their SE. For instance, gender stereotpyes often lead to women being regarded as less competent compared to men, in particular in the field of science, technology, engineering and mathematics (Niepel et al., 2019; Breda et al., 2020). Finally, our findings are in line with previous research showing a significant association between children’s age and their competencies, with older children outperforming younger ones (e.g., Niklas and Schneider, 2017).

However, in addition to parents’ beliefs and numeracy activities, there are other contributing factors which have a potential impact on children’s numeracy abilities. For instance, Puglisi et al. (2017) did not find a direct association of the informal home literacy environment with children’s literacy skills while controlling for parental factors. They argued that parents’ genetics may also be an important factor for passing on good numerical and mathematical skills onto children. A genetic component on children’s mathematical abilities was also mentioned by Hart et al. (2009), who analyzed data on 314 same-sex twins.

Genetic influences on children’s academic achievement are discussed in numerous studies (see e.g., Ludwig et al., 2013; Baron-Cohen et al., 2014; Davis et al., 2014; Pettigrew et al., 2015). Moreover, prior research also discussed intergenerational transmissions between parents and their children concerning mathematical abilities. Here for example, parents’ approximation number system was associated with toddlers’ number processing, even after controlling for children’s vocabulary and parents’ mathematical abilities (Navarro et al., 2018). Consequently, it is recommended to consider a genetically sensitive design when investigating children’s mathematical abilities in the context of the HNE (Napoli and Purpura, 2018; Hart et al., 2021) and a more differentiated look at parental factors and other influencing aspects is needed. Still, our findings contribute to the understanding of parental aspects that are associated with numeracy-related activities at home and children’s numeracy outcomes (Figure 2).

The fact that our findings are supported by the results and suggestions of previous research, but are also in contrast with some other research (e.g., Sonnenschein et al., 2012; del Río et al., 2017, 2019; Peacock-Chambers et al., 2017; Vasilyeva et al., 2018), underlines the importance of further investigation of parental factors, such as beliefs and home numeracy activities, but also of differences between mothers and fathers. In our view, surveys and questions assessing parents own beliefs and numeracy practices need to be discussed in the context of missing measurement invariance and the prevailing criticism of using self-reported data only in most studies (Missall et al., 2016; Zippert and Rittle-Johnson, 2020). Our findings implicate that we need to improve our measurement methods before investigating the potential differences between mothers and fathers. Further, we suggest that future research should not only take potential differences between mothers and fathers into account, but should also analyze how parents influence each other in their beliefs and activities and thus may together impact on the development of their children.

Further, clearer definitions of different aspects of parents’ beliefs are needed, as we noticed different wordings and definitions for similar items and scales in research. For example, del Río et al. (2020) used the term “parents’ self-concept” when using very similar items and questions that we defined as parental self-efficacy. Additionally, other relevant family characteristics (e.g., children’s and parent’s sex, age, SES etc.) need to be considered and examined concerning their associations with each other, as well as with different aspects of the HNE and children’s numeracy competencies (see Hornburg et al., 2021). We would like to stress the importance of developing novel surveys or of improving existing surveys to measure parental mathematical beliefs and the home mathematical environment and potential differences between parents.

Despite these open questions, our findings underline the need for more practical implications to support children’s early numeracy development. Here, interventions designed to further investigate gender-based differences, self-efficacy beliefs and HNE may improve our understanding and uncover mechanisms that are at work (see Kaya and Lundeen, 2010). For instance, during a parent evening and through playful parent-child activities at the kindergarten, parents can be made aware of the importance of the role their beliefs and actions play for their children’s competency development (Niklas et al., 2016). Another option would be to apply digital interventions, for example via a mobile app which regularly provides useful information or practical tips for parents that they may use in their everyday life together with their children (Niklas et al., 2020a).

Further, a long-term intervention program with early childhood teachers showed change and modifications in beliefs toward mathematics as well as in their pedagogical content knowledge (Bruns et al., 2017). Kaya and Lundeen (2010) reported an effective intervention in the context of parents’ attitudes and interest in science by applying an interactive home, school and community collaboration. Their findings showed that family interactions and parents’ attitudes toward science became more positive and the interest in the involvement of elementary science increased due to the intervention. Consequently, we assume that interventions including information on the HNE and gender-based differences, and enabling parents to inform themselves about these topics and share their thoughts, feelings and ideas in a group accompanied by a professional will lead to a better understanding and a potential change of their belief set. Such interventions may also influence parental abilities, attitudes, and feelings about mathematics, but more importantly might also improve the relationship with their children and positively impact on children’s beliefs and competencies.

### Limitations and Further Research

Our study has several limitations that need to be considered when interpreting these findings. First, we only used cross-sectional data, so that no causal interpretation of our findings is possible. Longitudinal data collection and using a mixed-method approach would allow a greater insight onto this topic. However, many of our results align with findings of recent research (Sonnenschein et al., 2012; del Río et al., 2017, 2019, 2020).

Second, due to missing data, we only were able to analyze data from a reduced sample (*N* = 160). There were minor differences between fathers who remained and fathers who dropped out in regard to the IOMA, which needs to be taken into account when interpreting the results.

Third, we could not establish scalar measurement invariance between mothers and fathers as stated in our discussion. Therefore, all comparisons between mothers and fathers need to be interpreted cautiously. However, our descriptive analyses still provide very important information and indicate that studies should consider the sex of both, parents and children. Here, new measurement instruments are needed that work similarly for mothers and fathers (see also Hornburg et al., 2021). In addition, we did not test for potential differences between the different language versions of our survey items and this needs to be considered when interpreting the results of families with a language background other than German.

Fourth, our IOMA scale showed a low internal inconsistency, which might be driven partly by the fact that it only included three items. Here, a more comprehensive assessment of IOMA would be helpful for a more reliable measure.

Fifth, we did not control for siblings of the study child. Consequently, the results for parents need to be interpreted with caution, as siblings of the same or different sex may also influence parental mathematical beliefs and activities as well as children’s numeracy competencies.

Sixth, our findings rely on self-reported data of parents only, which may lead to biased and social desirable answers (Missall et al., 2016; Zippert and Rittle-Johnson, 2020). Here, parent-child interactions captured by observational measures or qualitative data on more specific aspects and actions at home assessed through interviews may be useful additional methods of data collection.

In our research, we focussed on mothers and fathers as these were the most common main caregivers for the children in our sample. Here, we only assessed heteronormative families as our total sample included only one case that differed from the majority. However, it would also be of great interest to investigate whether the results would change for same-sex parents or other caregivers (e.g., grandparents).

Seventh, it should be mentioned that the current study did not use a genetically sensitive design. Future research should consider both environmental and genetic factors when investigating associations of children’s numeracy abilities and family characteristics.

Finally, it has to be mentioned that the sex of the children was reported as a binary construct in our study only which is consistent with the historical approach in this field but is questioned in latest research regarding its adequacy (see e.g., Berner et al., 2020). However, for the age group analyzed in our study, we still believe that a binary classification will be appropriate for almost all children.

## Conclusion

Our findings indicate that parents regard boys to be more competent in mathematics than girls. Additionally, parents’ self-efficacy differed with mothers showing a lower mathematical self-efficacy compared to fathers. Further, mothers’ mathematical self-efficacy and fathers’ reported importance of mathematical activities at home correlated with actual numeracy activities at home. Only the frequency with which fathers engaged in numeracy activities with their child were positively associated with children’s numeracy competencies.

Moreover our findings raise very important questions for the field of educational psychology: What do we measure when we assess mathematical beliefs and activities of one main caregiver via survey only? How would results differ when both main caregivers are surveyed with questionnaires that show scalar measurement invariance? Are the findings valid for both, boys and girls, or do we need to put a greater focus on parental and child sex differences from early age onward?

Our results indicate that we are still in need of better, standardized and thoroughly evaluated assessment tools (see also Hornburg et al., 2021). Further, more research on the various influencing factors and their interaction in the context of children’s numeracy competency development is needed. Our findings demonstrate that there may be relevant differences between mothers’ and fathers’ beliefs and numeracy activities at home, which need to be considered for a better understanding of children’s early numeracy development.

The main goal should be to support children’s competencies development regardless of the main caregiver’s and the child’s sex. Consequently, we also need more detailed information about existing differences and about how best to support children and their parents according to the individual needs of the child. Future research should consider and analyze practical implications which will provide more insight into topics such as HNE and beliefs toward mathematics and which will lead to parental awareness on the importance of the role their beliefs and actions play for their children’s competencies development.

## Data Availability Statement

The raw data supporting the conclusions of this article will be made available by the authors, without undue reservation.

## Ethics Statement

The studies involving human participants were reviewed and approved by the European Research Council Executive Agency and Ethics Committee of the Faculty of Psychology and Educational Sciences at the University of Munich. Written informed consent to participate in this study was provided by the participants’ legal guardian/next of kin.

## Author Contributions

AM and FN conceptualized the main ideas of the manuscript. AM conducted the main analyses and wrote the original draft. AM, AW, EB, and FN investigated the study, reviewed, and edited the manuscript. FN was responsible for the resources, supervision, project administration, and funding. EB was responsible for the data curation. All authors have read and agreed to the published version of the manuscript.

## Conflict of Interest

The authors declare that the research was conducted in the absence of any commercial or financial relationships that could be construed as a potential conflict of interest.

## Publisher’s Note

All claims expressed in this article are solely those of the authors and do not necessarily represent those of their affiliated organizations, or those of the publisher, the editors and the reviewers. Any product that may be evaluated in this article, or claim that may be made by its manufacturer, is not guaranteed or endorsed by the publisher.

## Footnotes

1 One single case of a mother-mother dyad was present in our sample. Consequently, this case had to be excluded for statistical reasons.
