# Supplementary material for: Associations Between Children’s Numeracy Competencies, Mothers’ and Fathers’ Mathematical Beliefs, and Numeracy Activities at Home
Source: Front Psychol. 2022 Apr 14;13:835433. doi: 10.3389/fpsyg.2022.835433 (PMC9048258; doi:10.3389/fpsyg.2022.835433)
Supplement: Supplementary file 1 [file Data_Sheet_1.pdf]

frontiers

Supplementary Material

Supplementary Data

Measurement invariance and factor loadings

In the second analytical step, we tested the measurement invariance of our full model for configural, metric and scalar invariance. Here, the change of the alternative Comparative Fit Index (CFI; ≤-.01) and root mean square error of approximation (RMSEA; ≤.015) was used instead of the very sensitive Chi-Square (χ^2) (Chen, 2007). For our planned analyses (e.g. *t*-tests, MGSEM) scalar invariance would be needed. However, our model only achieved configural invariance (see Table S1).

Table S1

Analyses of measurement invariance testing for the theoretical model.

Invariance	CFI	RMSEA	χ^2 (*df*)	χ^2	*p*	*Decision*
Configural	.98	.069	446.932 (254)			Accept
Metric	.96	.085	554.135 (270)	110.748	<.001	Reject
Scalar	.93	.103	910.978 (336)	497.373	<.001	Reject

Note. Fit Criteria for all measurement models CFI ≤ -.01; RMSEA ≤ .015.

Table S2

Factor loadings for mothers and fathers of the MGSEM

Construct	Items	Loadings of Mothers	Loadings of Fathers
NumC	MARKO-S	0.736**	0.738**
	Number sequences forwards	0.778**	0.778**
	Number sequences backwards	0.795**	0.798**
	Number symbol knowledge	0.839**	0.843**
	Knowledge of numerical representation	0.751**	0.751**
	Calculation	0.684**	0.674**
NA	Item 1	0.863**	0.836**
	Item 2	0.886**	0.835**
	Item 3	0.405**	0.429**
	Item 4	0.394**	0.383**
	Item 5	0.496**	0.460**
	Item 6	0.217*	0.273**
SE	Item 7	0.840**	0.797**
	Item 9	0.911**	0.894**
	Item 10	0.823**	0.922**
	Item 11	0.553**	0.923**
	Item 12	0.636**	0.931**
	Item 13	0.390**	0.820**
GS	Item 14	0.924**	0.902**
	Item 15	0.938**	0.945**
	Item 16	0.877**	0.804**

IOMA	Item 17	0.746**	0.551**
	Item 18	0.919**	0.806**
	Item 19	0.859**	0.627**

Note. Significance level ** *p* <.001, **p* <.01. See item description in Table S3 supplementary material.

Survey questions

The following tables show the relevant items of our parental survey assessing numeracy activities (Table S3) and parental beliefs towards mathematics (Table S4). Items 1 to 6 in Table S3 assess parents' NA at home. In Table S4, items 7 to 13 assess SE, items 17 to 19 assess GS, and the other items were used to assess parents' perceived IOMA at home.

Table S3

Parental survey - parents` numeracy activities at home.

Item	Description	Several times a week	Once a week	Every 2-3 weeks	Less often	Never
1	How often do you play counting games with your child (e. g., "Benjamin Blümchen: Lerne Zählen", "Die Maus Lern-Spiel-Sammlung", "Kosmolino: 1,2,3…")?	☐	☐	☐	☐	☐
2	How often do you play calculation games with you child (e. g., "Ich lerne Rechnen", "Zahlen und Rechnen", "Zählen und Rechnen mit Ernie und Bert", "1+2=3 Rechnen macht Spaß")?	☐	☐	☐	☐	☐
3	How often do you play dice games with your child (e. g., "Mensch ärger Dich nicht" or "Tempo, kleine Schnecke")?	☐	☐	☐	☐	☐
4	How often do you involve your child in shopping (e.g., weighing and counting groceries or paying at the checkout counter)?	☐	☐	☐	☐	☐
5	How often do you involve your child in cooking (e.g., counting, weighing, or measuring ingredients)?	☐	☐	☐	☐	☐
6	How often do you talk to your child about units of measurement (e.g., about weight, temperature, or speed)?	☐	☐	☐	☐	☐

Table S4

Parental Survey - Parental beliefs toward mathematics.

Item	Description	Not at all true	Rather not true	Neither true nor false	Rather true	Completely true
7	Mathematics is fun for me.	☐	☐	☐	☐	☐
8[1]	I avoid situations, in which I have to deal with mathematics.	☐	☐	☐	☐	☐
9	I am good at communicating mathematical content.	☐	☐	☐	☐	☐
10	I like communicating mathematical content.	☐	☐	☐	☐	☐
11	My mathematical skills are good (calculating total prices, measuring ingredients etc.).	☐	☐	☐	☐	☐
12	My advanced mathematical skills are good.	☐	☐	☐	☐	☐
13	In school, I was good at math.	☐	☐	☐	☐	☐
14	It is important to me that my child does mathematical activities at home.	☐	☐	☐	☐	☐
15	It is important to me to be able to help my child in mathematics.	☐	☐	☐	☐	☐
16	My child often sees me doing mathematical activities.	☐	☐	☐	☐	☐
17	Girls need less assistance than boys in mathematics.	☐	☐	☐	☐	☐
18	Girls are more competent in mathematics than boys.	☐	☐	☐	☐	☐
19	Math is more often the favourite subject of girls than of boys.	☐	☐	☐	☐	☐

[1] Item 8 was excluded from our analyses due to a response bias, as it was the only negatively worded item (see e.g., Corwyn, 2000).
